# Olive Oil Antioxidants

**DOI:** 10.3390/antiox11050996

**Published:** 2022-05-19

**Authors:** Catalina Alarcón-de-la-Lastra

**Affiliations:** Department of Pharmacology, Faculty of Pharmacy, Universidad de Sevilla, 41012 Sevilla, Spain; calarcon@us.es

Extra virgin olive oil (EVOO) is the typical source of fats in the Mediterranean diet. EVOO is considered the highest quality vegetable oil, but also implies a high sensory quality. The organoleptic properties are greatly affected by the incidence of certain factors, both intrinsic, such as the olive variety, and extrinsic, such as the growing conditions, so that each EVOO has a particular flavor. Furthermore, these flavors are susceptible to change under the influence of other factors throughout the oil’s shelf-life, such as oxidation or temperature. 

While fatty acids are essential for the EVOO’s nutraceutical properties, multiple biological activities are also due to the presence of polyphenols, such as phenol alcohols and acids, secoiridoids, lignans and flavones that are being actively investigated for their purported biological and pharma-nutritional properties in diseases with an important pathogenetic contribution of oxidative and peroxidative stress and damage, mainly cancer, autoimmunity, chronic inflammation, neurodegeneration, obesity, insulin resistance and diabetes, atherosclerosis and ageing-related disorders, mediated in part by direct antioxidant actions. Several mechanisms have been investigated, such as including the imbalance of the redox code via either preventive or radical-trapping antioxidants, their ability to quench different kinds of radicals, i.e., O_2_^•−^, ^•^NO_2_, HO^•^, HOO^•^ radicals, in addition to their ability to contribute to the enzymatic decomposition of ROS and organic hydroperoxides through such enzymes as glutathione peroxidases and superoxide dysmutases, which increase the interest of studying these types of bioactive compounds in depth.

In this Special Issue, Mousavi et al. [1] studied the most important bioactive compounds of olive fruit in 61 international olive cultivars during two consecutive seasons. A large variability was detected for each metabolite analysed. Total phenol content varied on a scale of 1–10 (3831–39,252 mg kg^−1^) in the cultivars studied. Squalene values fluctuated in an even wider range (1–15), with values from 274 to 4351 mg kg^−1^. Total sterols ranged from 119 to 969 mg kg^−1^, and total tocopherols ranged from 135 to 579 mg kg^−1^ in the fruit flesh. This work provided solid information on the fruit metabolite profile of a wide range of cultivars, which will facilitate the development of new olive genotypes through genomics-assisted breeding.

Another interesting article in this Special Issue, by Bidooki and coworkers [2], deals with squalene, one of the main components of virgin olive oil, which possesses natural antioxidant properties. To increase its delivery and enhance its actions, squalene was successfully encapsulated in PLGA NPs. Squalene protected AML12 liver cell lines against oxidative and endoplasmic reticulum stress in a Thioredoxin domain-containing 5 (TXNDC5). Squalene was also able to reduce ROS in AML12 cells and improve cell viability in ER-induced stress by decreasing Ern1 or Eif2ak3 expressions. The authors concluded that squalene protects mouse hepatocytes from oxidative and endoplasmic reticulum stress via several molecular mechanisms that depend on TXNDC5. 

The third full article of this Special Issue by Diaz-Montaña et al. [3], who studied the evolution of the oxidative process of extra virgin olive oil (EVOO) under mild storage conditions for 8 months, monitoring the individual content of 15 phenols and the changes in the phenolic profile of the nonflavoured oil compared to the same flavoured oil (rosemary and basil). Throughout storage, a reduction in the concentration of the phenols was detected, except for tyrosol and hydroxytyrosol. The addition of aromatic plants reduced the oxidative process, prolonging the shelf-life of the flavoured oil compared to the unflavoured oil.

Mancebo-Campos et al. [4] evaluated the oxidative effect of temperature on minor components (phenols, tocopherol, pigments) to design and develop a shelf-life prediction model. The kinetic behaviour of phenolic compounds, α-tocopherol and pigments during the storage of different virgin olive oil samples at different temperatures (25–60 °C) was evaluated. Hydroxytyrosol, tyrosol and α-tocopherol conformed to pseudo-zero-order kinetics, while secoiridoid derivatives of hydroxytyrosol and tyrosol, o-diphenols and total phenols apparently followed pseudo-first-order kinetics. Principal component analysis was used to transform the considered composition and degradation variables into a smaller number of uncorrelated principal components. In addition, multivariate linear regression was used to obtain several modelling equations for shelf-life prediction, considering the initial composition and easily determinable experimental variables in accelerated storage.

Pozzetti et al. [5] chemically characterized and selected autochthonous Tuscany EVOOs to obtain hydroalcoholic phytocomplexes, which were assayed to establish their anti-inflammatory and vasorelaxant properties. Apigenin and luteolin were found as representative flavones; other components were pinoresinol, ligstroside and oleuropein. The extracts showed anti-inflammatory and antioxidant properties via the modulation of NF-κB and Nrf2 pathways, respectively, and good vasorelaxant activity, both in the presence and absence of an intact endothelium.

Lammi et al. [6] studied the modulation of DPP-IV activity of two extra virgin olive oil (EVOO) phenolic extracts (BUO and OMN) using in vitro, cellular and in silico molecular modeling investigations. The in vitro DPP-IV activity assay showed a dose-dependent inhibition. Moreover, both BUO and OMN reduced the DPP-IV activity expressed by Caco-2 cells. The most abundant and representative secoiridoids, such as Oleuropein, oleacein, oleocanthal, hydroxytyrosol and tyrosol, reduced the DPP-IV activity. Finally, in silico molecular docking simulations permitted the study of the binding mode of these compounds. 

Oleocanthal (OLE), a characteristic and exclusive secoiridoid of the *Oleoaceae* family, is mainly found in extra virgin olive oil (EVOO). Since the pathogenesis of rheumatoid arthritis (RA) involves inflammatory and oxidative components, this study by Montoya et al. [7] was designed to evaluate the preventive role of dietary OLE-supplemented effects in a collagen-induced arthritis (CIA) murine model. Dietary OLE prevented bone, joint and cartilage rheumatic affections induced by collagen. Levels of circulatory matrix metalloproteinase (MMP)-3 and proinflammatory cytokines (IL-6, IL-1β, TNF-α, IL-17, IFN-γ) were significantly decreased in secoiridoid-fed animals. Additionally, dietary OLE was able to diminish COX-2, mPGES-1 and iNOS protein expressions, as well as PGE_2_ levels. The mechanisms underlying these protective effects could be related to Nrf-2/HO-1 axis activation and the inhibition of relevant signaling pathways, including JAK-STAT, MAPKs and NF-κB, thus controlling the production of inflammatory and oxidative mediators. Overall, these results exhibit preliminary evidence concerning OLE as a novel dietary tool for the prevention of autoimmune and inflammatory disorders, such as RA. 

Gutiérrez-Miranda et al. [8] studied the beneficial effects of oleacein (OLE), an olive secoiridoid, in an experimental model of experimental autoimmune encephalomyelitis (EAE). Treatment with OLE significantly reduced the clinical and histological signs typical of EAE by modulating the expression of pro- and anti-inflammatory cytokines and minimising the serum levels of anti-MOG35 antibodies. In addition, OLE significantly decreased the presence of oxidative system parameters, while increasing the ROS disruptor, Sestrin-3. Mechanistically, OLE prevented NLRP3 expression, the phosphorylation of p65-NF-κB and decreased the synthesis of proinflammatory mediators in BV2 cells without affecting the viability and phagocytic capacity of the BV2 microglia. In addition, OLE prevented oxidative stress factor-induced apoptosis of RGC-5. The authors conclude that OLE induces neuroprotective effects, through both antioxidant and anti-inflammatory effects, indicating this natural product as a candidate to be considered for the investigation of EAE treatments.

Reverón et al. [9] identified, at genome scale, the antimicrobial mechanisms of HXT action as well as molecular mechanisms that potentially enable *L. plantarum* to cope with the effects of hydroxytyrosol (HXT), one of the main and health-relevant plant phenolics present in olive oil by genome transcriptomic profiling. The transcriptomic profile revealed an HXT-triggered antioxidant response, involving genes from the ROS (reactive oxygen species) resistome of *L. plantarum*, genes coding for H_2_S-producing enzymes and genes involved in the response to thiol-specific oxidative stress. *L. plantarum* transcriptionally reprogrammed nitrogen metabolism and involved the stringent response (SR) to adapt to HXT, as indicated by the reduced expression of genes involved in cell proliferation or related to the metabolism of (p)ppGpp, the molecule that triggers the SR.

Rochetti et al. [10] detected a significant impact of in vitro gastrointestinal digestion on the polyphenolic profiles of different commercial extra-virgin olive oils (EVOO). In particular, the compounds which were found to be the most affected by the in vitro digestion were flavonoids (cyanidin and luteolin equivalents). In this regard, oleuropein-aglycone was converted to hydroxytyrosol during the pancreatic step. Taken together, the present findings corroborate the suitability of untargeted metabolomics coupled to in vitro digestion methods to investigate the bioaccessibility of phenolic compounds. 

The review article in this Special Issue by García-Oliveira et al. [11] discusses the evolution of flavors in EVOO shelf-life. The organoleptic properties related to the flavour of EVOO depend on the presence mainly of certain carbonyl-type components of the volatile fraction and of some minor compounds such as phenolics. However, these properties are conditioned by certain intrinsic factors, the olive variety in particular, and extrinsic factors, especially the growing conditions, so that each EVOO has a particular flavour. Moreover, these flavours are susceptible to modifications throughout the shelf-life of the oil, such as through oxidation or temperature changes. This paper describes some of the most notable compounds responsible for the unique flavour and aroma of EVOO, the factors that affect them, the mechanism that leads to the degradation of EVOO and how the flavours can be altered during the shelf-life of the oil, as well as several suggested strategies for the preservation of this flavour, on which the quality of the product also depends. 
